# Recent Progress in Exploring Dietary Nutrition and Dietary Patterns in Periodontitis with a Focus on SCFAs

**DOI:** 10.3390/nu17193150

**Published:** 2025-10-02

**Authors:** Jing-Song Mao, Hao-Yue Cui, Xuan-Zhu Zhou, Shu-Wei Zhang

**Affiliations:** Department of Periodontics, Liaoning Provincial Key Laboratory of Oral Diseases, School and Hospital of Stomatology, China Medical University, Shenyang 110002, China; 15736161718@163.com (J.-S.M.); 13238824056@163.com (H.-Y.C.); Z1305078@163.com (X.-Z.Z.)

**Keywords:** dietary nutrition, dietary pattern, SCFAs, periodontitis

## Abstract

Dietary patterns greatly affect periodontitis, a chronic inflammatory disease that compromises both dental and systemic health. According to the emerging evidence, periodontal risk is more strongly associated with the overall dietary quality, especially fiber density intake, than any one micronutrient. While the average intake in industrialized countries is only half of the recommended 30 g day^−1^, high-fiber diets such as the Mediterranean diet, the Dietary Approaches to Stop Hypertension (DASH), and whole-food plant-based diets are consistently associated with a 20–40% lower periodontitis prevalence. Dietary fiber plays a central role in regulating immune responses, strengthening tissue barriers, improving metabolic homeostasis, and shaping a healthy microbiome through its microbial fermentation products: short-chain fatty acids (SCFAs). This makes it a biologically rational and clinical evidence-supported strategy for the prevention and management of periodontitis. Integrating high-fiber diet recommendations into routine periodontal care and public health policies could be a crucial step towards more comprehensive oral and systemic health management. This narrative review elaborates on the mechanistic, observational, and intervention data highlighting the role of dietary fiber, especially SCFAs, in periodontal health.

## 1. Introduction

Periodontitis is a highly prevalent and costly inflammatory disorder that is estimated to affect more than 3 billion people in its mild form and over 1 billion in its severe form, including early-stage cases [1]. Studies have observed that a balanced diet has an essential role in maintaining periodontal health [2]. Decades of research on isolated micronutrients (e.g., vitamins C and D, and long-chain ω-3 fatty acids) have revealed limited periodontal benefits and failed to explain most of the risk variations in population studies. In contrast, modern research using dietary pattern indices such as the Dietary Inflammatory Index (DII), Mediterranean Diet Score (MDS), and Healthy Eating Index (HEI) have demonstrated that periodontal health is more strongly associated with overall diet quality than individual nutrients. These indices evaluate the variety, balance, and intake adequacy of consumed foods and nutrients. Among the protective dietary components, fiber consistently emerges as a key factor [3,4]. Studies have found that high-fiber diets can reduce the risk of periodontitis in a dose-dependent manner, with whole-grain fiber offering greater protection than soluble fiber from fruits [5,6,7]. Mendelian randomization (MR) analyses and prospective cohort studies have associated genetically proxied or self-reported high fiber intake with a reduced risk of periodontitis. The involved mechanisms are pleiotropic, including SCFA-mediated immunological control, modulation of the oral and gut microbiomes, attenuation of postprandial glucose and lipid spikes, and enhancement of epithelial barriers [8,9]. Compared to absolute intake, dietary fiber density holds greater predictive value. Cross-sectional analyses revealed a significant inverse correlation between dietary fiber density and periodontitis risk, which is primarily mediated through modulation of the oral microbiome, mitigating inflammatory responses, and maintaining metabolic homeostasis [10]. Clinical trials have demonstrated that supplementation with specific fibers (β-glucan, FOS, and RS4) improves periodontal outcomes, with mixed fermentable fiber blends (RS + inulin) yielding the optimal efficacy [11].

In this review, we discuss how dietary fibers affect periodontal health. We summarize how different fiber types interact with periodontal tissues and the underlying mechanisms. Drawing on clinical evidence, we also propose practical dietary guidelines for oral health alongside public health and policy recommendations.

## 2. Methods

A narrative literature search was conducted across PubMed, MEDLINE, ScienceDirect, and Web of Science using combinations of MeSH terms and free-text keywords relevant to periodontitis, dietary fibers, dietary patterns, SCFAs, periodontal tissues, and dietary strategies in oral diseases. The search included English-language articles published before September 2025. Relevant original research articles detailing human and animal studies, systematic reviews, meta-analyses, and review papers were included, and outdated publications and those lacking sufficient data to support the analysis were excluded.

## 3. Dietary Patterns Rich in Fibers and the Effects on Periodontal Condition

### 3.1. Mediterranean Diet

The Mediterranean diet (MD) provides 32–45 g of mixed dietary fiber per day [12]. Polyphenols such as hydroxytyrosol, oleuropein, and flavan-3-ols are naturally present in the fiber matrix and reach the colon where co-metabolism by butyrogenic bacteria releases phenolic acids and increases butyrate production by approximately 20%. Butyrate improves intestinal tight junction integrity and reduces endotoxemia. Additionally, MD consumption lowers plasma LPS-binding protein levels [13]. A systematic review found that the MD’s high arabinoxylan oligosaccharide (AXOS) content promotes *Bifidobacterium* enrichment, modifies the propionate-to-acetate ratio, enhances glucagon-like peptide-1 (GLP-1) secretion, and suppresses neutrophil chemotaxis [14].

An eight-week randomized controlled trial using MD-based menus increased daily fiber intake from 16 to 38 g, resulting in a 28% reduction in BOP and downregulation of *NF-κB* and *TLR4* gene expression in gingival tissues. Salivary cortisol levels also decreased, indicating an interaction between diet and stress modulation [12]. A long-term cohort study demonstrated that higher Mediterranean Diet Scores (MDSs) correlated with a reduced incidence of periodontitis, with fiber density mediating half of this protective effect [15].

### 3.2. DASH and Diets High in Fruits and Vegetables

The DASH pattern provides 29–36 g fiber daily, which is primarily composed of pectin, cellulose, and residual β-glucans [2]. High potassium can increase systemic alkalinity, regulating osteoclastic bone resorption. The rapid fermentation of soluble pectins generates propionate that interacts with free fatty acid receptor 3 (FFAR3) on sympathetic ganglia. This interaction reduces blood pressure and enhances gingival micro-vascular flow [16]. Additionally, nitrate-rich leafy greens in the DASH diet promote oral *Rothia* species that convert nitrate to nitrite, consequently increasing nitric oxide production and improving blood flow [17]. A six-week crossover study demonstrated that substituting one daily meal with a DASH-style salad increased fiber intake by 14 g, reduced probing depth by 0.4 mm, and shifted the subgingival microbial community toward health-associated taxa [18,19]. The top quartile of DASH adherence in national survey data showed markedly reduced odds of severe periodontitis, with fiber density mediating over half of this correlation [20,21,22]. These findings highlight the potential of the DASH diet as an adjunctive nutritional strategy for periodontal health management.

### 3.3. Whole-Food Plant-Based Regimes

Due to the large proportion of slowly fermentable, resistant starch from legumes, intact whole grains, and cooled tubers, a whole-food plant-based (WFPB) diet can increase fiber intake to 45–60 g per day [23]. Resistant starch fuels butyrogenic specialists in the distal colon, thereby conserving the mucosal energy supply and avoiding proteobacterial blooms. A greater systemic presence of SCFAs and a higher butyrate-to-acetate ratio in people adhering to a WFPB diet have been demonstrated by stable isotope tracer investigations [3,24].

The research indicates that a WFPB diet can aid in weight management, improve blood glucose control, and enhance cardiovascular health. These systemic health improvements may contribute to a reduced risk of periodontitis or slow its progression to some extent [25]. Individuals following a WFPB diet exhibit lower levels of systemic inflammatory markers, creating a more favorable environment for periodontal health [26].

### 3.4. Low-Carbohydrate/Ketogenic Diets

Very-low-carbohydrate ketogenic diets (VLCKDs) often reduce fiber intake below 10 g per day. Although ketone bodies exert anti-inflammatory effects, a recent meta-analysis of randomized clinical trials revealed that the early phases of a VLCKD can exacerbate gingival bleeding and halitosis due to fiber scarcity and dysbiosis [27]. Sequencing studies revealed declines in saccharolytic commensals and an increase in proteolytic anaerobes when fiber intake falls dramatically. Reduced SCFA production allows mucin-degrading microbes to flourish, which were shown to increase gut permeability and systemic LPS in a mouse model [28].

In a human study, there were no changes in periodontal parameters in the 20 generally healthy volunteers who followed a ketogenic diet for 6 weeks [29]. A scoping review included eight ketogenic diet studies, three of which suggested significant positive effects on periodontal health [30]. Therefore, there may be a positive correlation between long-term ketogenic diets and periodontitis. Although the evidence is inconsistent, the ketogenic diet has demonstrated anti-inflammatory effects but further research is needed [31,32].

Diets naturally rich in various fiber matrices, including the Mediterranean, DASH, and whole-food plant-based diets, have been associated with improved periodontal measurements through their effects on metabolic, microbial, and immunological regulatory pathways, and play a positive role in restoring periodontal tissue to a healthy state [33].

## 4. Biological Association Between Dietary Fiber and the Periodontium

### 4.1. Classification of Dietary Fiber

Dietary fiber (DF) is a type of carbohydrate polymer that resists digestion by human digestive enzymes in the small intestine but is fermented by gut microbiota in the large intestine [34]. The recommended daily fiber intake is 25 g for women and 38 g for men to maintain intestinal health and prevent chronic metabolic diseases [35]. The contemporary definition of dietary fiber includes non-carbohydrate lignin, resistant starches (RS types 1–5), non-starch polysaccharides, and related oligosaccharides with a molecular weight greater than 3 units. Researchers have found that dietary fiber and the gut microbiome are mutually beneficial to each other, and the health of the gut microbiome is closely related to immune responses, epithelial integrity, electrolyte reabsorption, and peripheral organ function [36]. The classification and the characteristics of the different types of fiber are presented in Table 1.

#### 4.1.1. Solubility Versus Matrix Entrapment

Water-soluble polymers include pectins, β-glucans, guar galactomannan, and inulin type fructans, while lignin, hemicellulose, and cellulose are among the insoluble frameworks [37]. From a periodontal perspective, a polymer’s solubility predicts its metabolic effects (post-prandial attenuation) whereas its insolubility affects oral shear: the mastication of wheat bran, chicory root fiber, or apple pomace generates plaque-disrupting forces and increases salivary flow by 50–100%, raising the bicarbonate buffering capacity that restrains proteolytic and acid-sensitive pathogens [5].

#### 4.1.2. Viscosity/Rheology

Viscous soluble fibers such as oat β-glucan (molecular weight > 200 kDa), psyllium arabinoxylan, and high-methoxyl pectin generate solution viscosities of 5–15 Pa·s during gastrointestinal shear [38]. By lowering the systemic glycemic variability, which can cause periodontal tissue oxidative stress, these fibers can slow chyme transit, lower glucose and chylomicron absorption, and indirectly modulate neutrophil oxidative burst [6].

#### 4.1.3. Fermentability and Gas Kinetics

Fructooligosaccharides (FOSs) exhibit fermentation half-lives of 2–30 h, whereas retrograded amylose (RS3) requires significantly longer fermentation periods [39]. While slowly fermentable celluloses provide distal substrates that prevent the “energy gap” that causes proteobacterial blooms and mucosal inflammation, highly fermentable fibers produce quick proximal colon SCFA peaks. Crucially, combinations of fast- and slow-fermenting fractions produce both distal epithelial trophic support and proximal butyrate production, a pattern reflected in ancestral high-fiber diets [40].

**Table 1:** Physicochemical classes of dietary fiber.

Physicochemical Class	Key Properties and Effects	Examples	Ref.
Soluble Fiber	Delays gastric emptying and reduces post-meal blood sugar and cholesterol levels	Pectins, β-glucans, guar galactomannan, and inulin-type fructans	[5]
Insoluble Fiber	Removes dental plaque, increases saliva production, and inhibits pathogens	Wheat bran, chicory root fiber, or apple pomace	[5]
Viscous Fiber	Forms a high-viscosity solution (5–15 Pa·s), which slows down the absorption of glucose and fat, and improves blood sugar control	Oat β-glucan (molecular weight > 200 kDa), psyllium arabinoxylan, and high-methoxyl pectin	[38]
Fermentable Fiber	Rapid fermentation can increase the content of short-chain fatty acids in the proximal colon, while slow fermentation can support distal microbiota	Fructooligosaccharide (FOS) is a highly fermentable fiber and resistant starch (RS3) is a slowly fermentable fiber	[40]

### 4.2. Short-Chain Fatty Acids (SCFAs)

Niche specialist consortia carry out fibrous catabolism. The initial deconstruction involves GH32 fructanases, pectate lyases, and endo-β-1,4-glucanases tethered to Sus-like outer membrane complexes (e.g., those in *Bacteroides thetaiotaomicron*) [41]. The released oligosaccharides are transported into periplasmic phosphorolytic pathways, generating acetyl-CoA and phosphoenolpyruvate (PEP).

SCFAs are produced via three main reductive paths The acetyl-CoA pathway is widespread and fuels peripheral tissues while serving as a carbon source for hepatic and osteoblastic lipogenesis [42]. The propionate pathway, which proceeds via methylmalonyl-CoA, is predominantly mediated by certain *Veillonellaceae* and *Bacteroides* spp. This pathway contributes to reduced cholesterol production and the regulation of hepatic gluconeogenesis. Another significant route is the butyryl-CoA pathway involving butyrate kinase and acetate CoA-transferase, which produces butyryl-CoA. When generated by *Faecalibacterium prausnitzii*, *Eubacterium rectale*, and *Roseburia* spp., butyrate is the most immunologically potent SCFA in T-cell regulation. *Anaerostipes caccae* and *Eubacterium hallii* consume the lactate produced by *Bifidobacterium adolescentis* to generate butyrate, thereby improving the SCFA profile and highlighting the importance of the community structure in regulating the SCFA stoichiometry. The peak colonic concentrations range from 80 to 120 mM; the luminal pH falls from 7.0 to 5.5, thereby limiting pathogens such *Escherichia coli* O157 and *Fusobacterium nucleatum* [43].

**Figure 1:**
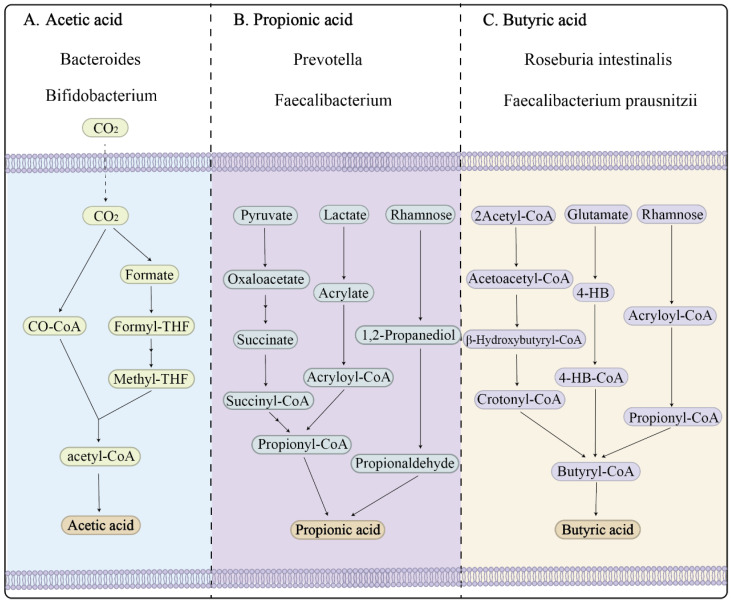
Schematic representation of major microbial pathways for short-chain fatty acid (SCFA) production. (**A**) Acetic acid production by *Bacteroides* and *Bifidobacterium*. (**B**) Propionic acid generation through pathways involving *Prevotella* and *Faecalibacterium*, including routes via succinate, acrylate, and propanediol. (**C**) Butyric acid synthesis by key butyrate-producing bacteria such as *Roseburia intestinalis* and *Faecalibacterium prausnitzii* via acetyl-CoA and glutamate metabolism.

The transport of short-chain fatty acids (SCFAs) to peripheral tissues is primarily achieved through three pathways (Figure 1). Under physiological pH conditions (SCFA pKa ≈ 4.8), approximately 1–3% of the SCFAs passively diffuse in their undissociated form. SCFAs can also be transported through monocarboxylate transporters (MCTs) [44]. MCT1 is highly expressed in periodontal ligament fibroblasts (PDLFs) and mediates the uptake of SCFAs by endothelial and immune cells. Additionally, transport can occur via G protein-coupled receptor (GPCR) signaling pathways [45]. Free fatty acid receptors (FFAR2/FFAR3) on the surface of epithelial and leukocyte cells can be activated by SCFAs. Gingival biopsy studies have shown that dietary fiber supplementation can triple the mRNA expression of FFAR2 and FFAR3 in mice. Short-chain fatty acid receptors (FFAR2 and FFAR3) regulate immune responses through different G protein-coupled signaling pathways [46]. When FFAR2 binds to Gαi/o protein, it reduces the intracellular cAMP level, thereby inhibiting the activation of the NLRP3 inflammasome. When FFAR3 binds to Gαq protein, it activates phospholipase Cβ (PLCβ), leading to an increase in the intracellular calcium ion concentration and subsequently promoting the transcription of the anti-inflammatory cytokine IL-10 through the CREB signaling pathway [43]. These two receptor-mediated cascades constitute an important molecular mechanism through which short-chain fatty acids regulate inflammatory responses. Butyrate also diffuses to nuclei and suppresses class I and IIa histone deacetylases, with an IC50 of 0.5 mM, thereby enabling acetylated histone H3 to occupy the FOXP3 promoter and transforming naive CD4^+^ cells into regulatory T cells that target inflamed gingiva [47]. Radiolabeling (^14^C acetate) investigations demonstrated tracer absorption, peaking at 30–60 min, into alveolar bone, periodontal ligaments, and salivary glands, consistent with direct SCFA–tissue contact [48]. However, periodontal pathogens can also produce butyrate during metabolism, promoting periodontal tissue inflammation and reactivation of latent viruses. Therefore, we should view butyrate as a double-edged sword and in a comprehensive manner.

## 5. Host–Microbe Crosstalk: Fiber-Driven Modulation of Oral and Gut Microbiomes

DF has the ability to promote beneficial bacteria and provides them with specific nutrients. They can also lower the pH by producing short-chain fatty acids to inhibit pathogenic bacteria and regulate the intestinal-related immune system, ultimately regulating the composition of the intestinal microbiota and its metabolites [16]. Fiber-driven modulation of the oral and gut microbiomes involves direct oral-site effects and systemic consequences [49,50]. Substrate competition occurs as low-molecular-weight FOS and galacto-oligosaccharides diffuse into saliva (≈0.2–0.5 g after a 10 g load), enabling *Streptococcus sanguinis* and *Rothia dentocariosa* (equipped with raffinose permeases and β-fructofuranosidases) to outcompete proteolytic *P. gingivalis* at adhesion loci [51]. Concurrently, arginine deiminase-positive commensals utilize oligosaccharides to fuel alkaline production (NH_3_), raising the plaque pH to 7.2 and restraining acid-tolerant pathogens [18]. Cross-kingdom interactions are evident as butyrate suppresses *Candida albicans* hyphal transition by inhibiting Hda1-mediated histone deacetylation, diminishing fungal–bacterial biofilm synergy. Systemically, high-fiber diets increase *Roseburia* and *Faecalibacterium* while reducing pathobionts like *Enterobacteriaceae*. This results in SCFA-driven skewing of bone marrow myelopoiesis towards Ly6C^−^ non-classical monocytes and IL-10-producing dendritic cells, which circulate to gingival tissues and blunt pro-inflammatory chemokine gradients [31]. Additionally, SCFA-induced claudin-1 and occludin transcription reinforce colonic tight junctions, reducing plasma LPS levels by 30–50%. Acetate and propionate further inhibit neutrophil oxidative burst, thereby attenuating collateral collagenase activation in the periodontal pocket [52].

## 6. Mechanistic Pathways Linking Fiber to Periodontal Homeostasis

### 6.1. Immune Modulation

#### 6.1.1. Regulatory T Cell Induction and Cytokine Re-Balancing

Diet-derived acetate, propionate, and butyrate activate FFAR2 (GPR43), FFAR3 (GPR41), and GPR109A on lamina propria dendritic cells and naïve CD4^+^ T cells. In animal models, receptor activation decreaes intracellular cAMP levels and activates PI3K–Akt and ERK1/2 at the same time, resulting in the nuclear localization of the acetyl-CoA pool and allosteric inhibition of class I histone deacetylases [53]. Specifically, at lysine 9 and lysine 27 of the FOXP3 promoter, histone H3/H4 hyperacetylation exposes the chromatin for STAT5 and Runx1 binding, therefore promoting de novo differentiation of peripheral FOXP3^+^ regulatory T cells [54]. In addition, butyric acid stabilizes the expression of FOXP3 by enhancing its acetylation, further strengthening the inhibitory function of Tregs [55].

#### 6.1.2. Neutrophil Chemotaxis, NET Formation, and Oxidative-Burst Control

Although neutrophils are necessary for bacterial control, they are the most abundant leukocytes in the gingival crevice. Their hyper reactivity accelerates tissue loss. Human polymorphonuclear neutrophils show strong expression of FFAR2. The activation of FFAR2 by SCFAs induces Ca^2+^ signal transduction and chemotaxis of neutrophils [56,57,58,59]. By lowering Rac2 GTP loading, propionate binding limits actin polymerization, thereby slowing chemotaxis toward IL-8 gradients inside the junctional epithelium.Butyrate lowers NETosis through two different pathways: (i) HDAC inhibition prevents PAD4-mediated histone citrullination, which is necessary for chromatin decondensation, and (ii) lower mitochondrial ROS generation suppresses NADPH oxidase assembly [60]. In vitro, 1 mM butyrate reduces PMA-triggered NET release by 40% yet preserves the intracellular killing of *P. gingivalis*, showing targeted regulation rather than immunosuppression. Hyperglycemic spikes produce advanced glycation end products (AGEs) that engage neutrophil RAGE, reducing neutrophil priming. This also helps to further minimize oxidative bursts. A 35 g daily preload can lower post-prandial glucose spikes by 25% and within two weeks, the levels of circulating cell-free DNA (a surrogate NET marker) decrease [61].

### 6.2. Barrier Integrity and Connective Tissue Preservation

#### 6.2.1. Reinforcement of Epithelial Tight Junctions

The first host barrier against subgingival infections is formed by gingival and junctional epithelia. However, butyrate is a high energy source for these cells and triggers AMPK α1 phosphorylation at Thr172. Through Sp1 acetylation, AMPK phosphorylates ZO 1 and occludin, stabilizing them at the apicolateral membrane and simultaneously increasing claudin 1 transcription. By inducing Nrf2 translocation, SCFAs stimulate antioxidant proteins (HO 1 and NQO1), protecting tight junctions from ROS-driven breakage. Rats fed a resistant starch diet demonstrated better gingival microvascular perfusion using laser Doppler flowmetry, indicating a better supply of oxygen and the nutrients necessary for epithelium turnover [62,63].

#### 6.2.2. Collagen Synthesis and Matrix Metalloproteinase Inhibition

Type I and III collagen are synthesized by periodontal ligament fibroblasts (PDLFs) for Sharpey fiber regeneration. However, butyrate-mediated HDAC inhibition increases TGF β/Smad signaling, enhancing COL1A1 and COL3A1 gene expression. Furthermore, SCFA exposure boosts the activities of lysyl oxidase and prolyl 4 hydroxylase, enzymes essential for triple helix stability and cross-link development. Matrix metalloproteinases have the ability to dissolve collagen in periodontal tissues and play a crucial role in the pathogenesis of periodontal diseases [64]. Propionate increases TIMP 1 and TIMP 3 expression, balancing the levels of MMP 2, 8, and 9 that are up regulated by *P. gingivalis* LPS [43]. Ultimately, by restricting peroxynitrite-mediated breakage and AGE cross-linking, the lower systemic and topical oxidative nitrosative stress contribute to protecting collagen [65,66].

### 6.3. Metabolic Regulation: Glycemic Control and Insulin Signaling

**Figure 2:**
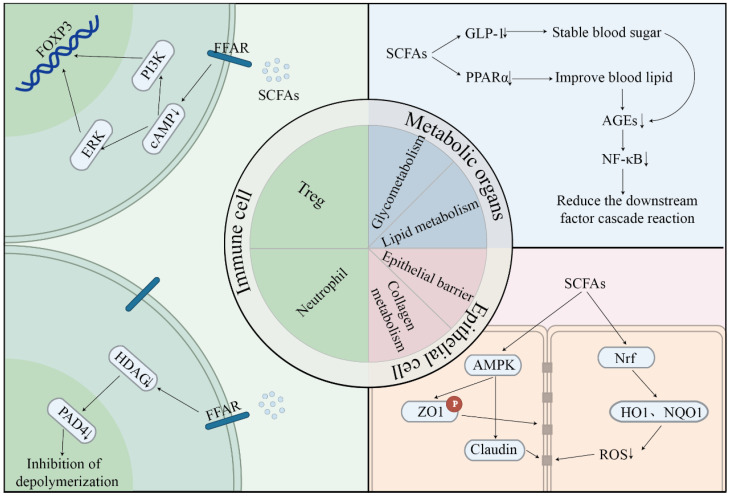
Three pathways through which dietary fiber influence periodontal homeostasis. Immune modulation: SCFAs promote Treg differentiation via HDAC inhibition and FOXP3 acetylation, suppressing Th17 responses and reducing IL-17A levels. Neutrophil chemotaxis and NETosis are tempered via FFAR2 and ROS suppression. Barrier integrity: Butyrate enhances tight junction proteins (ZO-1, occludin, and claudin) via AMPK activation and Nrf2-mediated antioxidant defense (HO-1 and NQO1), improving epithelial barrier function. Metabolic regulation: SCFAs improve glycemic control via GLP-1 release and AMPK/GLUT4 activation, reducing AGEs and NF-κB signaling. ↓—indicates that the molecule or reaction is downregulated under a specific action.

Highly viscous fibers (oat β-glucan, psyllium, and guar gum) create 5–15 Pa·s gastric gels, slow down stomach emptying, and increase chyme viscosity, thereby reducing glucose flow into enterocytes. This is reduced after prandial glycemic peaks and delays SGLT 1-mediated absorption [67]. By acting on endocrine pathways, SCFAs enhance metabolic benefits: propionate and acetate increase L cell production of GLP 1 and PYY, enhancing first-phase insulin secretion and prolonging satiety, thereby preventing excess caloric intake [68]. Acetate increases intracellular acetyl-CoA in hepatocytes, which allosterically inhibits pyruvate carboxylase, thus regulating gluconeogenesis. SCFAs activate AMPK and increase GLUT4 translocation in skeletal muscle (Figure 2), improving glucose elimination. Reduced glycemic variability lowers AGE generation, and reduced AGE–RAGE interaction in gingival tissues lowers NF-κB activation and the downstream cytokine cascades [69].

As described above, dietary fiber acts as a master regulator of the periodontal ecosystem by coordinating immune tolerance, strengthening the epithelial and connective architecture, and recalibrating systemic metabolism, transforming a state of chronic catabolic inflammation into one of balanced homeostasis and active tissue repair [70].

## 7. Limitations of Current Evidence and Knowledge Gaps

The current evidence linking dietary fiber and periodontitis has several limitations and knowledge gaps. Measurement errors are a concern, as most cohorts rely on FFQs that systematically underreport fiber intake (particularly fermentable oligosaccharides) by 10–20%, with biomarker-calibrated exposure data rarely being reported. Residual confounding factors persists, for example, high-fiber consumers could differ in other behaviors (e.g., smoking, oral hygiene, and healthcare use). Propensity-scored re-analyses can atteunate these effects but cannot eliminate associations. Heterogeneous clinical endpoints (mixing CPI, CDC/AAP, self-reports, and radiographic thresholds) complicate meta-analysis, and intervention trials vary in terms of the baseline therapy. Most RCTs are short term (<12 weeks) and small (<100 participants) and lack long-term data on tooth loss or implant survival. Fiber subtypes (insoluble/soluble, viscous/non-viscous, and RS subtypes) are rarely parsed separately, making mechanistic attribution difficult. A significant trans-ethnic evidence gap exists, with high-burden regions (South Asia and sub-Saharan Africa) lacking prospective studies despite divergent microbiomes and diets. Causal mediation via microbiome shifts (e.g., SCFA production) remains inferential, as only three trials integrated dietary fiber intake measurements with subgingival metagenomics and host transcriptomics. Finally, while the benefits plateau above 35 g per day, the dose–response relationship, potential upper limits (≥50 g may impair mineral absorption/tolerance), optimal blend, and ceiling for periodontal benefits are undefined.

## 8. Future Research Directions

### 8.1. Long-Term Pragmatic Trials with Tooth Loss Endpoints

Future studies have to go beyond proxy periodontal assessments like probing depth and clinical attachment loss to produce definitive, patient-centered results, including tooth loss. With durations beyond five years, pragmatic trials could produce the required longitudinal data to convincingly prove that dietary fiber can be used as a preventative intervention. Diverse populations reflecting the real-world heterogeneity should be enrolled in these studies that take into account the varying baseline fiber intakes, genetic predispositions, oral hygiene practices, socioeconomic levels, and degrees of health literacy. Robust randomization and blinded assessments of dental endpoints will enable the identification of the ideal fiber dosages, sources, and regimens required to significantly slow down periodontal disease progression [71]. Furthermore, to clarify the mechanisms underlying the effects of dietary fiber intake on clinical outcomes, concurrent evaluations of systemic biomarkers (glycemic indicators, inflammatory cytokines, and SCFAs) and microbiome alterations (oral and gut bacteria) should be conducted.

### 8.2. Multi-Omics Mapping of Fiber–Microbe–Host Interactions in the Periodontium

An integrated multi-omics strategy is needed to disentangle the complicated interaction among dietary fiber consumption, microbial dynamics, and host responses in periodontal tissues. The changes within dental biofilms, gingival tissues, serum, saliva, and gastrointestinal samples should be mapped using high-throughput sequencing methods and metagenomic, transcriptomic, metabolomic, and proteomic investigations. These approaches will reveal the changes in inflammatory, immunological, and oxidative stress pathways, identify the microbial taxa linked to fiber metabolism, and determine the metabolites that control host–microbe interactions [72]. Combining host transcriptomic and proteome studies with metabolic transcriptome studies of microbial communities will help to elucidate the degree to which dietary fiber alters periodontal pathogenesis at the molecular and cellular levels. These multi-omic profiles will not only offer deeper mechanistic insights, but they can also be used to find predictive biomarkers for treatment response and disease prognosis, thereby guiding precision dietary strategies in periodontal therapy [18].

### 8.3. Precision Nutrition: Microbiome-Guided Fiber Prescriptions

Customized dietary plans based on microbiome markers show potential given the significant inter-individual diversity in microbiota composition and metabolic capability. Artificial intelligence and machine learning approaches should be used in future studies to link clinically obtained microbiome biomarkers with responsiveness to particular dietary fibers. To generate prediction algorithms, studies could combine specific metabolomic analyses (e.g., SCFAs, bile acids, and polyphenol metabolites) with next-generation sequencing of stool and saliva microbiota. These instruments could stratify people into phenotypic categories based on their responsiveness to fiber types including resistant starch, beta-glucan, inulin, or guar gum. Later, randomized controlled studies could confirm these microbiome-guided recommendations and iteratively improve the algorithms to maximize the therapeutic impact. By customizing fiber-based therapies to maximize the therapeutic benefit for every patient, this precision nutrition paradigm could greatly improve periodontal therapy and improving treatment adherence and efficacy [73].

### 8.4. Development of Functional Foods Targeting Oral Health

Promoting periodontal prophylaxis calls for the creation and validation of functional food products, especially those that provide therapeutic levels of dietary fiber, while also improving consumer acceptance [74]. Functional foods enhanced with resistant starch, β-glucans, guar gum, fructooligosaccharides, and polyphenols can offer practical ways for targeting the oral and gut microbiomes on a daily basis; these foods could include RS-4 fortified breads, fiber-rich beverages (smoothies or fortified yogurts), pulse-based snack meals, and fiber–polyphenolic-loaded confectionaries. The development of these food should ensure that these functional foods are delicious, reasonably priced, stable, and culturally relevant for the many different customer groups [75]. Clinical efficacy studies should also carefully evaluate the periodontal results, microbial changes, systemic biomarkers, and patient adherence measures to build evidence-based claims for these foods. The effective translation of these breakthroughs from the laboratory to the market will depend on cooperation between academics, the food industry, and public health organizations.

## 9. Conclusions and Clinical Recommendations

The collective evidence from mechanistic, intervention, and observational studies have highlighted dietary fiber’s multifunctional role in periodontal health. The epidemiological evidence has repeatedly linked high fiber intake and adherence to fiber-rich diets to a lower incidence and severity of periodontitis. Strong biological effects of dietary fiber including systemic anti-inflammatory, metabolic, and microbiota-modulating actions—mediated mostly by gastrointestinal fermentation and consequent SCFA generation—have been demonstrated. In particular, in integrated systemic health paradigms, this data strongly supports dietary fiber as a reasonable supplementary approach in controlling periodontal disease.

Clinically, the inclusion of dietary fiber into periodontal therapy requires simple, pragmatic methods for patient education at assessments. Dentists and hygienists should aggressively check for low fiber intake and offer tailored dietary guidance and use dietary adjuncts (ω-3 PUFA, polyphenols, and vitamin D) to maximize the therapeutic effects. However, several practical considerations must be addressed to ensure safety and adherence.

Potential Adverse Effects and Intolerance: Rapidly increasing fiber intake, particularly with highly fermentable fibers (FODMAPs), may induce gastrointestinal discomfort (bloating, flatulence) in some individuals, especially those with irritable bowel syndrome (IBS). A gradual introduction of fiber and guidance toward well-tolerated sources (e.g., oat bran, psyllium) is recommended.

Mineral Interactions: Very high fiber intake (≥50 g/day) may impair the absorption of minerals such as calcium, iron, and zinc due to high phytic acid content in whole grains and legumes. This risk can be mitigated by advising diverse fiber sources, proper food preparation (e.g., soaking and fermentation), and ensuring adequate mineral intake.

Supplementation in WFPB Diets: For patients adhering to strict whole-food, plant-based (WFPB) diets, attention should be paid to potential deficiencies in vitamins B12 and D, calcium, and zinc. Routine monitoring and appropriate supplementation are advised to support overall and periodontal health.

To facilitate clinical application, a concise set of recommendations for periodontists and hygienists is summarized in Table 2.

Public health policies should include fiber enhancement in more general nutritional programs; support staple food reformulation and better health labels that incorporate fiber and periodontal outcomes; and customize interventions to address the cost constraints of fiber-rich diets. Future periodontal care paradigms have to acknowledge dietary fiber not only as a complement but also as a fundamental component of therapeutic and preventive programs. Including fiber-centric dietary recommendations into regular periodontal treatment links clinical practice with modern nutritional science and provides a route towards better oral and systemic health results.

## Figures and Tables

**Table 2:** Take-home recommendations for integrating dietary fiber into periodontal care.

	Key Actions for Clinicians	Examples and Targets
Assessment	Routinely screen for dietary fiber intake when taking patient history.	Ask about daily fruit, vegetable, legume, and whole-grain consumption. Target: 30–38 g per day.
Counseling	Advise a gradual increase in fiber intake from diverse food sources. Emphasize the benefits for oral and systemic health.	Suggest adding one serving of legumes, berries, or whole grains per day; encourage adequate water intake.
Meal Examples	Provide simple, practical meal ideas to help patients achieve the daily target.	Breakfast: Oatmeal with berries and nuts (10 g fiber) Lunch: Salad with leafy greens, chickpeas, and quinoa (15 g of fiber) Dinner: Stir-fried vegetables with brown rice and lentils (13 g of fiber)
Monitoring and Follow-up	Schedule follow-ups to assess tolerance and adherence. Address GI symptoms if they arise.	Re-evaluate dietary habits at periodontal maintenance visits (3–6 months). Adjust recommendations based on individual tolerance.

## Data Availability

No new data were created or analyzed in this study. Data sharing is not applicable to this article.
